# JAK/STAT Signaling in Oral Lichen Planus: From Cytokine-Driven Immune Dysregulation to Barrier Dysfunction and Targeted Therapy

**DOI:** 10.3390/cimb48101006

**Published:** 2026-09-30

**Authors:** Alessandro Polizzi, Roberto Campagna, Giovanni Tossetta, Paolo Pesce, Giorgia Maria Marmo, Marco Mascitti, Gaetano Isola

**Affiliations:** 1 Department of General Surgery and Surgical-Medical Specialties, School of Dentistry, University of Catania, 95123 Catania, Italy; giorgiamarmo284@gmail.com (G.M.M.); gaetano.isola@unict.it (G.I.); 2 Department for the Promotion of Human Science and Quality of Life, San Raffaele Roma University, Via di Val Cannuta, 247, 00166 Rome, Italy; roberto.campagna@uniroma5.it (R.C.); giovanni.tossetta@uniroma5.it (G.T.); 3 Department of Surgical Sciences and Integrated Diagnostics (DISC), University of Genoa, 16132 Genoa, Italy; paolo.pesce@unige.it; 4 Department of Clinical Specialistic and Dental Sciences, Marche Polytechnic University, 60126 Ancona, Italy; m.mascitti@staff.univpm.it

**Keywords:** oral lichen planus, JAK/STAT signaling, JAK1, JAK2, JAK3, TYK2, STAT1, STAT3, epithelial barrier, Janus kinase inhibitors

## Abstract

Oral lichen planus (OLP) is a chronic, immune-mediated condition in which the oral epithelium acts simultaneously as a target of cytotoxic injury and a driver of persistent mucosal inflammation. This narrative review examines Janus kinase/signal transducer and activator of transcription (JAK/STAT) signaling as a network of cell- and cytokine-dependent signaling modules rather than a single linear pathway. Human OLP tissues show elevated expression of JAK1, phosphorylated JAK1, and various STAT-family proteins across both epithelial and inflammatory compartments. Functional modules demonstrate that IFN-γ–JAK1/JAK2–STAT1 sensitizes basal keratinocytes to cytotoxic attack, while JAK2/STAT3 regulates epithelial survival, tight-junction integrity, and E-cadherin/CD103-dependent intraepithelial lymphocyte retention. Additionally, metabolic and redox inputs converge on the STAT3–CCL5 axis to reinforce T-cell recruitment, with spatial analyses placing cytotoxic T cells directly at the epithelial interface. Although clinical responses to targeted JAK inhibitors (including JAK1, JAK1/2, JAK1/3, JAK3/TEC, and TYK2 agents) are encouraging, most current evidence remains observational. Because inhibitor selectivity is relative, therapeutic efficacy alone cannot identify the primary upstream cytokine driver. Advancing OLP management requires moving beyond broad pathway inhibition toward spatially resolved phospho-endotyping linked with standardized clinical phenotypes, mechanistic biomarkers, and targeted local therapies to enable a true precision treatment strategy.

## 1. Introduction

Oral lichen planus (OLP) is a chronic inflammatory disorder of the oral mucosa with a pooled prevalence close to 1%, although estimates vary substantially with geography, diagnostic criteria and sampling strategy [1,2]. Clinically, the disease spans asymptomatic reticular or plaque-like lesions to painful erythematous and erosive forms that may fluctuate for years, relapse after treatment withdrawal and materially impair oral function and quality of life [3,4,5].

The apparently straightforward label “oral lichen planus” conceals an important methodological problem: clinical and histopathological criteria do not always converge, and oral lichenoid lesions or lichenoid epithelial dysplasia may enter mechanistic series under an OLP diagnosis [6,7,8,9,10]. This matters because molecular studies performed on diagnostically heterogeneous cohorts can create the illusion of biological inconsistency when part of the heterogeneity is nosological. Accordingly, a pathway-centered review must distinguish direct OLP evidence from findings derived from broader lichen planus or lichenoid disease.

The traditional pathogenic model describes antigen-specific or autoreactive T cells targeting basal keratinocytes and producing an interface mucositis. That model remains useful, but it is too static to explain why lesions recur at the same sites, why erosive disease can behave differently from reticular disease, or why keratinocytes continue to shape inflammation after the initiating antigen remains unidentified [11,12,13,14]. More recent work instead portrays OLP as a spatially organized epithelial–immune ecosystem in which cytotoxic and helper T-cell subsets, dendritic cells, stromal cells and keratinocytes exchange cytokines and chemokines across a structurally compromised interface [15,16].

JAK/STAT signaling is particularly suited to organize this complexity because four Janus kinases and seven STAT proteins translate extracellular cytokine identity into rapid, cell-dependent transcriptional programs. Crucially, this does not mean that OLP is a “JAK/STAT disease” in the singular: the biological meaning of pathway activation depends on which cytokine receptor is engaged, which JAK pair is available, which STAT is phosphorylated and in which cell compartment the signal is received [17,18,19,20]. In this sense, JAK/STAT signaling is better understood as a set of cell- and cytokine-dependent modules, in which the same pathway can generate distinct biological outputs in keratinocytes, T cells, or other immune cells according to the upstream cytokine and cellular context. This distinction also tempers therapeutic interpretation. Repeated clinical responses to JAK/TYK2 inhibitors indicate pathway tractability, but they do not prove that one cytokine branch dominates every lesion [21,22,23,24]. The low but established risk of malignant transformation provides an additional reason to separate inflammatory control from oncologic surveillance; JAK/STAT activation should therefore not be portrayed as a proven carcinogenic driver in OLP, and suppression of inflammation cannot substitute for long-term mucosal surveillance [25,26,27,28,29].

Despite the growing number of studies implicating JAK/STAT signaling in lichen planus, the available evidence remains fragmented across cytokine profiling, tissue expression studies, experimental models and early therapeutic reports. What is still lacking is an integrated framework capable of linking receptor-level cytokine signaling to the spatial organization of the immune–epithelial interface and, ultimately, to treatment response. Such a synthesis is particularly relevant in OLP, where the same pathway may produce different biological effects depending on the cellular compartment, local cytokine milieu and epithelial state. Accordingly, the aim of this review is to critically examine the current evidence for JAK/STAT signaling in OLP, with particular emphasis on pathway architecture, keratinocyte–immune cell crosstalk, epithelial barrier dysfunction, disease endotypes and emerging therapeutic strategies.

## 2. Materials and Methods

This narrative review was developed through targeted searches of PubMed, Scopus, Web of Science and Google Scholar up to August 2026, complemented by backward and forward reference chaining from key mechanistic studies, clinical reports and recent reviews. Search concepts combined “oral lichen planus” or “lichen planus” with JAK, STAT, interferon, IL-6, IL-21, TYK2, keratinocyte, epithelial barrier, tissue-resident memory, chemokine and the names of currently reported JAK/TYK2 inhibitors. No lower publication-date limit was applied, allowing the inclusion of both foundational studies and recent mechanistic and translational evidence. Priority was given to human OLP tissue studies and OLP-specific functional models. Cutaneous or multisite lichen planus data were retained only when they clarified pathway causality, cell state or pharmacodynamic effects that have not yet been directly tested in oral mucosa. Studies were excluded when they did not provide information directly relevant to JAK/STAT signaling, OLP immunopathogenesis, JAK/TYK2-targeted therapy, or when they concerned unrelated oral or inflammatory conditions without clear mechanistic relevance to OLP. Because this was a narrative rather than a systematic review, study selection was guided by mechanistic relevance and contribution to the conceptual synthesis rather than by a formal predefined screening protocol.

## 3. General Architecture of JAK/STAT Signaling

The mammalian JAK/STAT system comprises four Janus kinases (JAK1, JAK2, JAK3 and TYK2) and seven STAT transcription factors—STAT1, STAT2, STAT3, STAT4, STAT5A, STAT5B and STAT6. JAKs are non-receptor tyrosine kinases constitutively associated with cytokine receptors that lack intrinsic kinase activity, whereas STATs are latent cytoplasmic transcription factors that convert receptor-proximal phosphorylation events into nuclear gene regulation. JAK1 and JAK2 are broadly used by multiple receptor families, JAK3 is most closely associated with common γ-chain cytokine receptors, and TYK2 has a prominent role in type I interferon and IL-12/IL-23 signaling [17,18].

Specificity is imposed largely by receptor–ligand architecture. IFN-γ signals primarily through JAK1/JAK2–STAT1, whereas type I interferons use JAK1/TYK2 with STAT1/STAT2. IL-6-family cytokines signal through gp130-associated JAKs with prominent STAT3 activation; common γ-chain cytokines (IL-2, IL-4, IL-7, IL-9, IL-15 and IL-21) predominantly use JAK1/JAK3 and recruit STAT3, STAT5 or STAT6 according to ligand and cell type. IL-12 and IL-23 share TYK2/JAK2-dependent receptor components but preferentially drive STAT4- and STAT3-centered programs, respectively [17,18]. These assignments are preferential rather than exclusive, because receptor abundance, signal duration and pathway crosstalk can reshape downstream output. Conversely, TNF-α and IL-17, although highly relevant to inflammatory networks, do not signal through canonical JAK-associated receptors.

## 4. JAK/STAT Signaling in OLP: From Receptor Architecture to Tissue Evidence

### 4.1. Cytokine Routing, Selectivity and Feedback Control

Type I and type II cytokine receptors lack intrinsic kinase activity and instead recruit JAK1, JAK2, JAK3 or TYK2 to their cytoplasmic domains. Ligand-induced receptor reorganization activates the associated kinases, generates phosphotyrosine docking sites, recruits STAT proteins and permits STAT phosphorylation, dimerization and nuclear translocation [17,18]. The canonical sequence is simple enough to diagram, but its biological output is not. Receptor stoichiometry, ligand concentration and duration, relative JAK and STAT abundance, chromatin state and concurrent MAPK, PI3K–AKT–mTOR or NF-κB activity can substantially reshape the transcriptional response [19,30].

For this reason, pharmacological “selectivity” is best interpreted at the level of signaling modules rather than drug labels. JAK1 participates in multiple receptor complexes by pairing with JAK2, JAK3 or TYK2, so a JAK1-predominant inhibitor can suppress several cytokine programs simultaneously; conversely, allosteric TYK2 inhibition preferentially modifies type I interferon and IL-12/IL-23 signaling while sparing many common γ-chain responses [31,32,33]. This modular view is particularly relevant to OLP, where IFN-γ, IL-6, IL-21, IL-23 and other cytokines coexist within the same tissue and may dominate different cellular compartments or disease phases [34,35,36,37,38].

Pathway persistence is also governed by negative feedback. SOCS proteins, PIAS proteins and protein tyrosine phosphatases limit signal amplitude and duration, while receptor internalization and cellular phosphatase activity further constrain repeated activation [17,18]. These brakes have received far less attention in OLP than upstream cytokines, creating a notable blind spot: chronic disease may reflect not only excess ligand but also defective signal termination or altered cellular responsiveness. The observation of nuclear pJAK1 in basal and parabasal OLP keratinocytes is therefore intriguing, but should be treated as a hypothesis-generating sign of possible non-canonical behavior rather than evidence of a defined nuclear JAK1 program [39].

### 4.2. What Is Directly Demonstrated in OLP Tissue?

The strongest direct tissue-level evidence for JAK activation in OLP comes from a 2026 retrospective study showing significantly higher JAK1 and phosphorylated JAK1 in both epithelial cells and the inflammatory infiltrate compared with normal oral mucosa [39]. Earlier work had already identified increased JAK3 and altered STAT2, STAT4 and STAT6 expression in oral mucosal disease, while a preliminary OLP cohort showed a stepwise rise in STAT3 signal from normal mucosa to reticular and erosive disease [40,41]. Together these studies establish pathway presence and activation, but they do not yet provide a coherent phosphoproteomic map. Antibody choice, phosphorylation site, tissue compartment and scoring method vary sufficiently that apparent agreement should not be overinterpreted.

The most persuasive advance has come from functional models rather than expression alone. In lichen planus keratinocytes, IFN-γ enhances cell-mediated cytotoxicity through JAK2/STAT1, directly linking an effector cytokine to target-cell vulnerability [42]. In an OLP-like mouse model, IFN-γ signaling and cytotoxic CD8+ T cells were required for the phenotype, local IFN-γ priming intensified disease through MHC-I upregulation, and baricitinib or ruxolitinib reduced disease burden [43]. A separate OLP-specific three-dimensional model linked JAK2/STAT3 activity to epithelial proliferation and apoptosis, E-cadherin/CD103-dependent intraepithelial lymphocyte homing and tight-junction integrity [44].

These convergent findings support a shift from asking whether “JAK/STAT is activated” to asking which JAK/STAT module is active, in which cell and with what tissue consequence. Table 1 summarizes the major cytokine–receptor–JAK–STAT combinations relevant to OLP while explicitly separating canonical signaling assignments from the strength of OLP-specific evidence [34,45,46,47,48]. The distinction is important for mediators such as IL-17, which is highly relevant to OLP but signals primarily through Act1/TRAF-dependent NF-κB and MAPK pathways rather than as a canonical JAK ligand; its relationship to JAK/STAT is indirect, through Th17 differentiation and network crosstalk [48,49,50].

A further limitation is the architecture of the available evidence. Most human studies are cross-sectional and compare OLP with normal mucosa, a design that is appropriate for detecting pathway differences but less informative for disease specificity. Future studies should include oral lichenoid lesions, inflammatory non-lichenoid controls and, where ethically justified, dysplastic lesions so that JAK/STAT activation can be separated from generic epithelial inflammation or tissue injury [6,8,10,26]. Equally important, total protein abundance should be paired with phosphorylation state, subcellular localization and cell-type assignment. Without these layers, an increase in a pathway component may reflect cellular recruitment rather than activation within resident cells. This distinction is central if JAK/STAT markers are to become therapeutic biomarkers rather than descriptive histological correlates.

## 5. The Immune–Epithelial Interface as the Relevant Biological Unit

### 5.1. Spatial Organization and Persistent T-Cell States

The histological signature of OLP, a dense band of lymphocytes immediately beneath the epithelium showing basal-cell degeneration, creates a highly constrained signaling geography. Cytokines are released within micrometers of basal keratinocytes, while antigen-presenting cells, stromal cells and microvascular changes influence which leukocytes gain access to the interface and how long they remain there [13,14,60,61]. This spatial constraint helps explain why bulk tissue or salivary cytokine measurements can be biologically informative yet mechanistically incomplete: the same absolute concentration may have very different consequences if its source is a tissue-resident cytotoxic T cell adjacent to the basement membrane rather than a dispersed inflammatory cell in the lamina propria [15,46].

Older descriptions of Th1/Th2 imbalance captured an important component of OLP but underestimate the diversity now documented within lesional T-cell populations. Th1, Th17, regulatory T-cell and Th9 phenotypes coexist, and their proportions vary with clinical context and disease phenotype [54,62,63]. The IL-23/IL-17 axis and lesional IL17A+ cells support Th17/Tc17 participation, whereas FOXP3+ regulatory cells indicate that suppressive programs are present but insufficient to restore tissue homeostasis [49,50,64]. The resulting lesion is therefore better understood as a competing cytokine network than as a binary polarization state.

Single-cell RNA sequencing and spatial transcriptomics have sharpened this model by identifying CD8+ tissue-resident memory (Trm) cells enriched near the basement membrane, particularly in erosive OLP. These cells showed IFN-γ, TNF-α and IL-17 programs consistent with repeated local effector activity and were associated with erosive behavior [52]. Independent characterization of intraepithelial lymphocytes identified CD4+CD8αα+ cells as a prominent epithelial population regulated by ThPOK and Runx3 [55]. Stromal fibroblast populations may contribute to the same geography through chemokine release, including CCL5, which can reinforce CD4+ T-cell recruitment [65].

### 5.2. Keratinocyte Signaling, Lymphocyte Homing and Barrier Failure

Keratinocytes are not passive victims of a cytotoxic attack. They respond to IFN-γ and other inflammatory cues by changing antigen presentation, chemokine secretion, adhesion molecules, survival pathways and checkpoint-related signals; in doing so, they determine how visible the epithelium becomes to T cells and how efficiently those cells are recruited and retained [45,66,67,68]. Basal-cell apoptosis remains a defining histological event, but the surrounding epithelial response is active and adaptive rather than simply degenerative [69,70].

The clearest example of this bidirectional behavior is the E-cadherin/CD103 axis. CD103-expressing intraepithelial lymphocytes can be retained through interaction with epithelial E-cadherin, while tight-junction proteins such as ZO-1 and occludin maintain the physical separation between the epithelial surface and the inflammatory compartment [44,55]. In the OLP three-dimensional homing model, JAK2/STAT3 inhibition reduced lymphocyte migration and increased ZO-1 and occludin expression, linking a signaling pathway to both immune-cell positioning and barrier phenotype [44].

This relationship suggests a self-reinforcing loop. Barrier disruption may increase exposure to salivary, microbial and mechanical stimuli; persistent lymphocyte residency can then sustain cytokine release at the same epithelial interface; and cytokine-driven keratinocyte responses further modify adhesion, chemokine output and susceptibility to cytotoxic injury. The consequence is not merely “inflammation plus epithelial damage,” but a tissue circuit in which each compartment alters the behavior of the other [15,44]. Metabolic inputs can feed into the same loop: ASCT2-mediated glutamine uptake promotes ROS-dependent STAT3 activation and CCL5 production in epithelial cells, while related metabolic programs influence Th1 differentiation [59,71].

The JAK2/STAT3 homing study also illustrates why pathway effects should not be reduced to a simple anti-inflammatory narrative. Inhibition decreased keratinocyte proliferation and increased pro-apoptotic signals while simultaneously reducing lymphocyte homing and improving barrier-protein expression [44]. Those outputs may be beneficial in an experimental setting in which aberrant proliferation and immune retention are linked, yet they also show that epithelial “restoration” is not synonymous with enhanced epithelial survival. The therapeutic meaning of STAT3 inhibition will therefore depend on disease stage, epithelial state and the balance between limiting pathological survival and preserving mucosal integrity [44,69].

**Figure 1:**
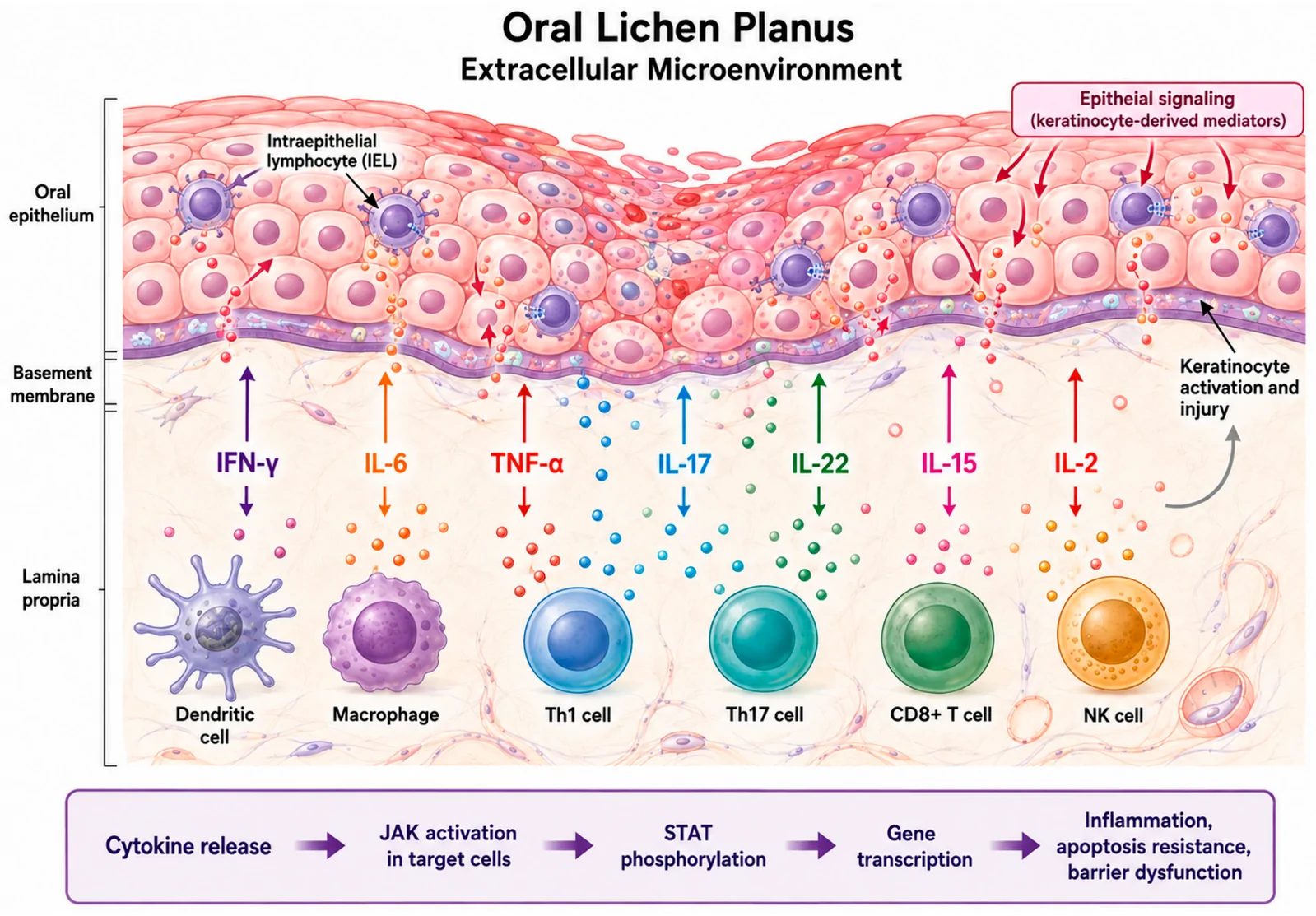
JAK/STAT signaling in the OLP microenvironment: extracellular overview. The scheme places oral keratinocytes, the basement membrane and inflammatory cells within the lesional architecture and illustrates cytokine flux toward the epithelial interface. Mucosal intraepithelial lymphocytes are also represented within the epithelial compartment, while keratinocytes are depicted as active contributors to the local inflammatory microenvironment through epithelial signaling and mediator release. The lower sequence summarizes the conceptual route from cytokine release to JAK activation, STAT phosphorylation and transcriptional effects. Cytokine-to-JAK coupling is context specific; IL-17 and TNF-α are shown as interacting inflammatory signals rather than canonical JAK ligands. Cytokine positions are schematic and should not be interpreted as exclusive source-cell assignments.

Figure 1 summarizes this extracellular architecture. It should be read as a spatial map of the OLP microenvironment rather than as a claim that every displayed mediator signals directly through JAKs; TNF-α and IL-17 are included because they interact functionally with JAK/STAT-regulated networks, not because they are canonical JAK ligands [15,72].

## 6. Functional JAK/STAT Modules and Network Crosstalk in OLP

### 6.1. IFN-γ–JAK1/JAK2–STAT1: Priming the Epithelial Target

Among the mechanistic pathways implicated in OLP, the IFN-γ–JAK1/JAK2–STAT1 axis is supported by substantial experimental and translational evidence. IFN-γ is repeatedly detected in OLP-associated cytokine profiles and increases epithelial MHC class I expression, thereby enhancing the capacity of cytotoxic T cells to recognize and attack basal keratinocytes [35,51]. In lichen planus keratinocyte models, IFN-γ increases cell-mediated cytotoxicity through JAK2/STAT1, providing a direct molecular bridge between cytokine exposure and target-cell vulnerability [42].

The OLP-like mouse model substantially strengthens this argument because disease severity depended on IFN-γ, local IFN-γ priming intensified lesions and JAK1/2 inhibition reduced the phenotype [43]. The model cannot reproduce the full heterogeneity or chronicity of human OLP, but its transcriptomic similarity to human lesions makes it more than a generic inflammatory oral model. It provides a platform in which a pathway can be perturbed before and after lesion induction and in which tissue-level outcomes can be related to defined immune mechanisms.

Cross-phenotype evidence is also informative. Cutaneous lichen planus shows a dominant IFN-γ/IL-21 transcriptional signature, and a mechanistic phase II study of baricitinib demonstrated rapid suppression of interferon-related programs together with reduction in a cytotoxic CXCL13+CD8+ T-cell population near the epidermal interface [37,73]. These data do not prove identical biology in oral mucosa, but they reinforce a general principle: interferon-high lichen planus may represent a pharmacologically tractable inflammatory state, and OLP trials should test that state directly rather than assume it from clinical morphology.

### 6.2. STAT3-Centered Circuits: IL-6, Metabolism, Chemokines and Barrier Biology

STAT3 occupies a more ambiguous position than STAT1. IL-6 is detectable in OLP tissue, saliva and serum, and canonical gp130 signaling can engage JAK1/JAK2/TYK2 to activate STAT3 [34,36]. In tissue, STAT3 signal rises from normal control to reticular and erosive OLP in a preliminary cohort, suggesting an association with clinical phenotype [41]. Yet total STAT3 abundance, Ser727 phosphorylation and canonical Tyr705 activation are not interchangeable measurements. A critical synthesis must therefore resist treating every increase in “STAT3” as equivalent evidence of one pathogenic mechanism.

Functional studies nevertheless show that STAT3 can influence biologically important epithelial outputs. JAK2/STAT3 inhibition in an OLP-specific model altered keratinocyte proliferation and apoptosis, reduced intraepithelial lymphocyte homing and increased ZO-1 and occludin expression [44]. A separate in vitro study reported attenuation of inflammatory and oxidative-stress features through a JAK–STAT3-related mechanism after GLP-1 exposure [53]. Taken together, these findings suggest that STAT3 should be viewed as a context-sensitive integration node for survival, repair and inflammatory programs rather than a uniformly pro-inflammatory transcription factor.

Metabolic studies extend this concept beyond receptor-proximal cytokine signaling. OLP T cells display enhanced mTOR–glycolytic activity, and metabolic manipulation changes proliferation, apoptosis and Th17/Treg balance [74]. In epithelial cells, ASCT2-mediated glutamine uptake increases ROS-dependent STAT3 activation and CCL5 production, facilitating T-cell infiltration; related work links glutamine uptake to Th1 differentiation through a ROS–EGR1–PAC1 pathway [59,71]. Salivary oxidative imbalance provides a clinical correlate of this redox-active environment [75].

The resulting picture is mechanistically important because it explains why two lesions with similar morphology may not be molecularly equivalent. One lesion may be dominated by interferon-driven STAT1 activity, another by a STAT3/CCL5/barrier circuit, and a third by overlapping cytokine and metabolic inputs. Such heterogeneity is a stronger rationale for biomarker-guided therapy than for indiscriminate pathway blockade.

### 6.3. JAK1/JAK3 and TYK2-Dependent Programs: Persistence, Type I Interferons and Th1/Th17 Plasticity

IL-2, IL-15 and IL-21 share the common γ-chain and rely primarily on JAK1/JAK3, with STAT5 or STAT3 as dominant downstream outputs. These cytokines are well positioned to sustain clonal expansion, cytotoxic-cell survival, memory and helper-cell differentiation [18,56]. OLP-specific evidence is strongest for the broader cytokine milieu and T-cell states rather than direct kinase-level mapping, so the common γ-chain module remains biologically plausible but incompletely resolved [34,54]. JAK3 overexpression in lichenoid tissue and clinical response to JAK3/TEC inhibition provide translational support without identifying which γ-chain cytokine is dominant in a given lesion [76,77].

Type I interferons provide a parallel JAK1/TYK2–STAT1/STAT2 route. Dendritic-cell involvement and altered STAT2 expression in OLP support an interferon-responsive environment, but the relative contribution of type I interferons versus IFN-γ remains unresolved [40,60]. This distinction is experimentally accessible because TYK2-selective pharmacology can perturb type I interferon and IL-12/IL-23 signaling without reproducing the broader profile of a JAK1 inhibitor.

IL-12 and IL-23 share IL-12Rβ1 and TYK2 but diverge toward STAT4- and STAT3-centered programs, making TYK2/JAK2 a plausible bridge between Th1/Tc1 and Th17/Tc17 inflammation [17,18]. OLP lesions show increased IL-23/IL-17 activity, and therapeutic targeting of Th17/Tc17 biology has improved lichen planus in proof-of-concept work [49,78]. Clinical responses to deucravacitinib are therefore mechanistically coherent, but they cannot establish whether type I interferon, IL-12, IL-23 or a combination of these TYK2-dependent signals was the decisive upstream driver [57,79].

The unresolved contribution of individual cytokine branches is therefore an experimentally testable feature of the model. JAK/TYK2 inhibitors perturb networks rather than isolated signals, and their clinical effects may reflect different dominant nodes in different patients.

### 6.4. Crosstalk with NF-κB, Cell-Death Programs and Epigenetic Regulation

JAK/STAT signaling cannot be interpreted in isolation from other inflammatory pathways. TNF-α is abundant in OLP but activates NF-κB and MAPK rather than canonical JAK receptors, and clinicopathological studies link NF-κB activation to TNF-α expression in atrophic–erosive and reticular disease [80]. In keratinocytes, combined TNF-α/IFN-γ stimulation produces chemokines that are attenuated when both NF-κB and JAK/STAT signaling are interrupted, illustrating how parallel pathways can cooperate at inflammatory gene promoters [72]. Such redundancy offers a plausible explanation for incomplete or transient responses to selective JAK inhibition.

Cell death adds another layer of complexity. Basal-cell apoptosis is well documented in OLP, but more recent work implicates caspase-1-associated inflammation and keratinocyte pyroptosis linked to metabolic disturbance [69,70,81,82]. STAT1 can increase cytotoxic susceptibility, whereas STAT3 may support epithelial survival or repair depending on context. Consequently, “reduced inflammation” and “reduced cell death” should not be assumed to move in parallel in every epithelial compartment.

Epigenetic and post-transcriptional regulation may further determine pathway sensitivity. Methylation changes involving E-cadherin and p16INK4a have been described in OLP/oral lichenoid lesions, and comparative tissue studies identify alterations in E-cadherin and stress-associated proteins [83,84]. miR-124/STAT3 regulation has been implicated in mesenchymal–stromal immunomodulation, while altered microRNA regulation of IL-22 provides another example of post-transcriptional control [58,85]. These findings remain fragmented and should be considered exploratory until integrated epigenomic, transcriptomic and phosphoproteomic analyses are performed in the same well-characterized lesions.

**Figure 2:**
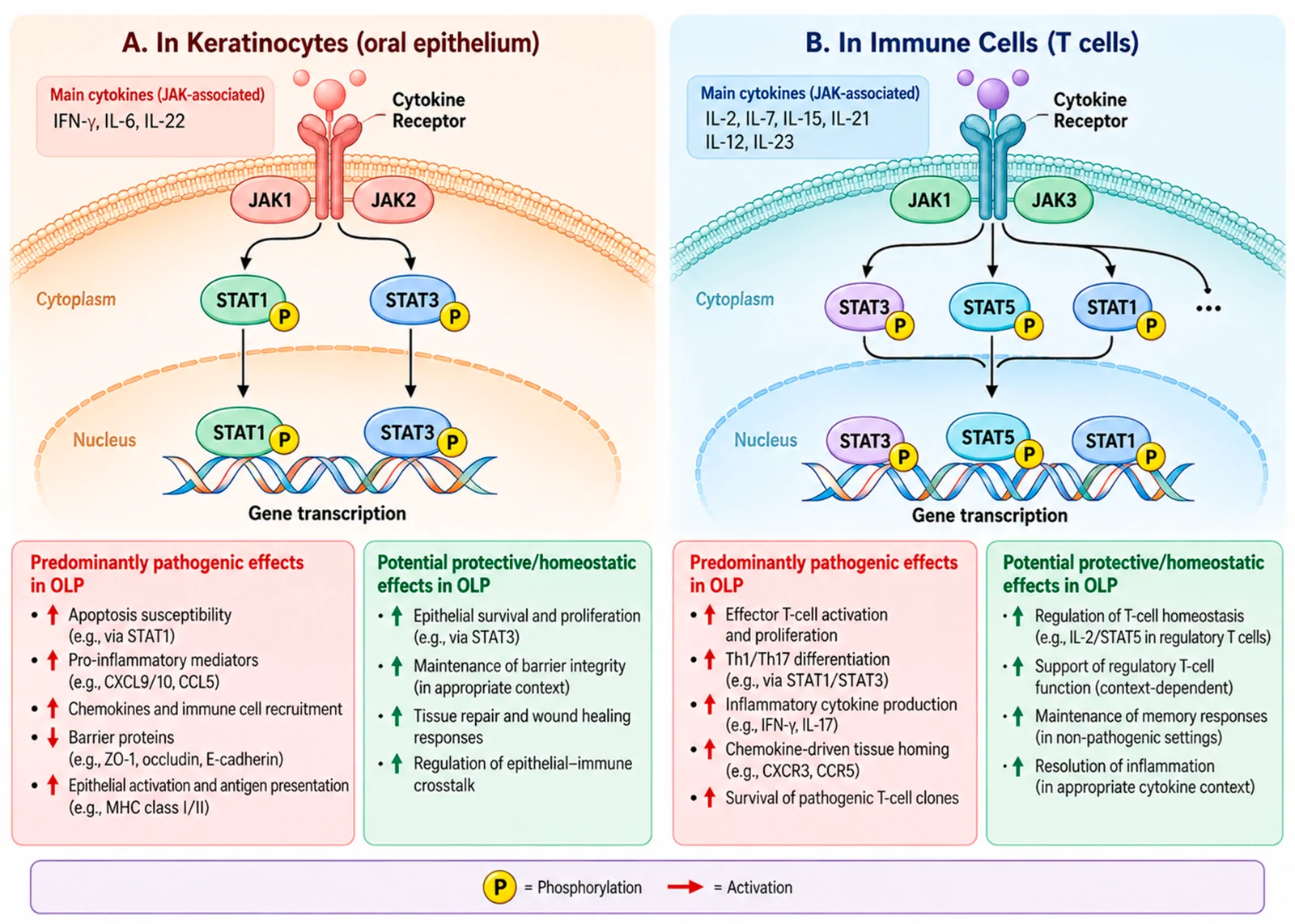
Intracellular JAK/STAT signaling in keratinocytes and immune cells. The two-compartment schematic illustrates how different JAK/STAT modules can produce distinct biological outputs in epithelial cells and T cells. In keratinocytes, STAT1- and STAT3-centered programs influence cytotoxic susceptibility, chemokines, survival and barrier proteins; in T cells, JAK1/JAK3- and related modules regulate proliferation, differentiation, persistence and cytokine production. For clarity, downstream effects are grouped as predominantly pathogenic or potentially protective/homeostatic in OLP; these categories are context-dependent and are not mutually exclusive. The figure is conceptual rather than an exhaustive receptor map.

Figure 2 summarizes the intracellular logic of the system by separating keratinocyte and T-cell signaling. That separation is not cosmetic: the same inhibitor can reduce epithelial chemokine output, interfere with T-cell differentiation and alter survival pathways at the same time, so the clinical response is a composite pharmacodynamic readout rather than a direct assay of one molecular branch [19,44].

## 7. From Pathway Expression to Clinical Endotypes

The central translational question is not whether JAK/STAT proteins can be detected in OLP, but whether pathway measurements improve biological stratification beyond conventional clinical categories. Increased JAK1/pJAK1 in epithelial and inflammatory compartments provides a strong starting point, yet the retrospective design and lack of matched downstream phospho-STAT profiling limit causal interpretation [39]. STAT3 differences between reticular and erosive lesions and reported abnormalities of JAK3, STAT2, STAT4 and STAT6 are similarly suggestive but methodologically heterogeneous [40,41].

Erosive OLP may be particularly informative because spatial studies suggest that it is not simply a more intense version of reticular inflammation. Enrichment of CD8+ Trm cells with IFN-γ, TNF-α and IL-17 programs at the epithelial interface indicates a distinct spatial immune state, while older work on Th9/Th17 and Th1/Th2 heterogeneity reaches the same general conclusion from different methodological directions [47,52,63]. A clinically useful endotype should therefore integrate cell identity, anatomical location and pathway activity—for example, basal epithelial pSTAT1/pSTAT3 with an interferon-response signature and Trm-associated markers—rather than rely on a single immunohistochemical signal.

A practical biomarker panel for early trials could combine pJAK1, pSTAT1, pSTAT3, epithelial MHC-I, CCL5, E-cadherin, ZO-1/occludin and CD8/Trm markers, with saliva used for serial cytokine or chemokine measurements when repeated biopsy is impractical [39,44,46]. The critical requirement is longitudinal validation: a biomarker that distinguishes lesions cross-sectionally but does not predict flare, durability of response or relapse after withdrawal will have limited precision-medicine value.

Clinical outcome measurement must advance in parallel. The REU score captures reticulation, erythema and ulceration and correlates with pain, while the OLP Investigator Global Assessment was developed to standardize symptomatic lesion severity for trials and practice [86,87]. Biomarker programs should be judged against these clinical instruments, not in isolation. Molecular complexity is useful only if it explains meaningful clinical heterogeneity or improves treatment decisions.

Sampling itself is a potential source of bias. A biopsy taken from the edge of an erosion contains a different mixture of basal keratinocytes, intraepithelial lymphocytes and inflammatory cells than a reticular plaque or an ulcer bed, and treatment can further shift that composition before a molecular assay is performed [44,52]. For this reason, future biomarker studies should predefine biopsy location, lesion morphology and recent topical therapy, and whenever possible pair tissue with standardized photography and spatial annotation. Otherwise, a putative molecular endotype may partly represent where the biopsy was taken rather than what biologic program characterizes the patient.

## 8. Therapeutic Translation of JAK/STAT Biology

### 8.1. Clinical Evidence Across JAK and TYK2 Inhibitors

The therapeutic literature has expanded rapidly and now spans several pharmacological profiles. Upadacitinib has the largest OLP-specific case-report footprint, with reports describing substantial improvement in erosive disease, sometimes within days to weeks [88,89,90,91,92,93]. Abrocitinib offers a JAK1-predominant strategy with direct OLP experience, while baricitinib combines OLP case evidence with strong mechanistic support from the IFN-γ-driven model and a pharmacodynamic lichen planus trial [43,73,94,95,96].

Tofacitinib provided early proof-of-concept for severe lichen planus and has subsequently shown substantial improvement in oral erosive disease in small case series [97,98,99]. Ritlecitinib, which preferentially inhibits JAK3 and TEC-family kinases, produced rapid improvement in a refractory erosive OLP case and is especially interesting because it perturbs a signaling branch distinct from JAK1/2-focused agents [77]. Ruxolitinib has the strongest OLP-specific support in preclinical work, whereas topical cutaneous lichen planus data provide a proof-of-principle for local JAK1/2 inhibition rather than direct evidence for oral delivery [43,100].

TYK2 inhibition is mechanistically attractive because deucravacitinib targets a narrower set of upstream cytokine programs than ATP-competitive pan-JAK strategies. A case report and a small erosive OLP series describe clinical benefit, providing a translational signal that type I interferon and/or IL-12/IL-23 biology may be therapeutically relevant [57,79]. The evidence is nevertheless too small to rank TYK2 against JAK1, JAK1/2 or JAK1/3 strategies, and cross-agent comparisons based on uncontrolled reports would be misleading.

Collectively, these reports show that the pathway is pharmacologically tractable across more than one kinase profile, but they do not establish comparative efficacy. Reviews and systematic syntheses appropriately emphasize the predominance of case reports, case series and small nonrandomized studies [21,22,23,24]. The next evidentiary step is therefore not simply to accumulate more successful cases, but to design studies in which diagnostic certainty, prior therapy, co-treatment, lesion phenotype, validated outcomes and pharmacodynamic biomarkers are prospectively standardized.

Publication bias is an additional, often underappreciated limitation. Dramatic responses in refractory OLP are more likely to be reported than partial responses, treatment failures or discontinuations, and many reports differ in background corticosteroid use, follow-up duration and criteria for relapse [21,22]. The current literature should therefore be read as evidence that several JAK/TYK2 nodes are clinically actionable, not as an estimate of response probability. Negative and neutral prospective data will be essential to determine whether the apparent breadth of efficacy reflects true pathway convergence or selective publication of successful cases.

### 8.2. What Therapeutic Response Does (and Does Not) Prove

Clinical improvement after JAK inhibition is often interpreted as confirmation that a particular JAK/STAT axis drives the disease. That conclusion is usually too strong. Because each inhibitor modifies several receptor pathways and more than one cellular compartment, response demonstrates pathway dependence at the network level, not the identity of a single causal cytokine [19,32]. Even apparently selective compounds can produce broader biological effects once tissue exposure and partner kinases are considered.

This caveat is particularly important when extrapolating from cutaneous lichen planus to OLP. The phase II baricitinib study and topical ruxolitinib experience provide high-value mechanistic and pharmacodynamic information, but the oral mucosa differs in barrier structure, microbial exposure, salivary clearance and malignant-surveillance requirements [73,100]. Oral efficacy should therefore be demonstrated directly rather than inferred from skin response.

Dose and route also complicate mechanistic interpretation. A systemic dose sufficient to suppress multiple cytokine pathways may produce a different biological signature from a topical formulation that reaches keratinocytes more efficiently than deeper immune populations. Concomitant corticosteroids or calcineurin inhibitors can further obscure attribution in case reports. Trials that include pharmacokinetic and pharmacodynamic sampling will be more informative than drug-versus-placebo comparisons alone because they can distinguish target failure from inadequate tissue exposure or pathway redundancy [19,32,100].

Table 2 maps the OLP-relevant therapeutic evidence by target and evidence type. The purpose is not to recommend one drug over another, but to show where clinical signals are strongest and where mechanistic interpretation remains speculative [21,22].

### 8.3. Safety, Oral-Specific Surveillance and Local Delivery

Systemic JAK inhibition carries recognized class concerns, including serious infection and herpes zoster, cytopenias, lipid and hepatic abnormalities, and warnings regarding cardiovascular events, venous thromboembolism and malignancy in selected high-risk populations [101,102,103]. These risks cannot be transferred numerically from rheumatoid arthritis cohorts to generally younger dermatologic or oral-medicine populations, but they must shape patient selection, baseline assessment and longitudinal monitoring.

OLP adds oral-specific considerations. Chronic erosions, candidiasis risk, periodontal or odontogenic infection, tobacco and alcohol exposure, and pre-existing dysplasia all affect the risk–benefit calculation. Most importantly, clinical suppression of inflammation does not abolish the need to biopsy persistent, indurated or changing lesions and to maintain long-term cancer surveillance [25,27,104]. A published case in which upadacitinib-treated erosive lichen planus was complicated by oral squamous cell carcinoma is not evidence that the drug caused the carcinoma, but it is a useful reminder that therapeutic success and oncologic vigilance are separate clinical objectives [104].

The accessibility of the oral mucosa creates a practical opportunity for local delivery. A mucoadhesive or carefully formulated topical JAK inhibitor could generate high lesional exposure while reducing systemic exposure, an approach conceptually supported by topical ruxolitinib activity in cutaneous lichen planus and by selective topical JAK1 work in other interface inflammatory disorders [100,105]. Salivary clearance, lesion topography and mucosal permeability remain important formulation challenges, but they do not preclude a local therapeutic strategy. More recent evidence has also strengthened the case for local pathway inhibition: a 2026 systematic review of topical JAK inhibitors across the lichen planus spectrum reported promising clinical activity and favorable short-term tolerability, although oral-specific formulation and efficacy data remain limited [106].

**Figure 3:**
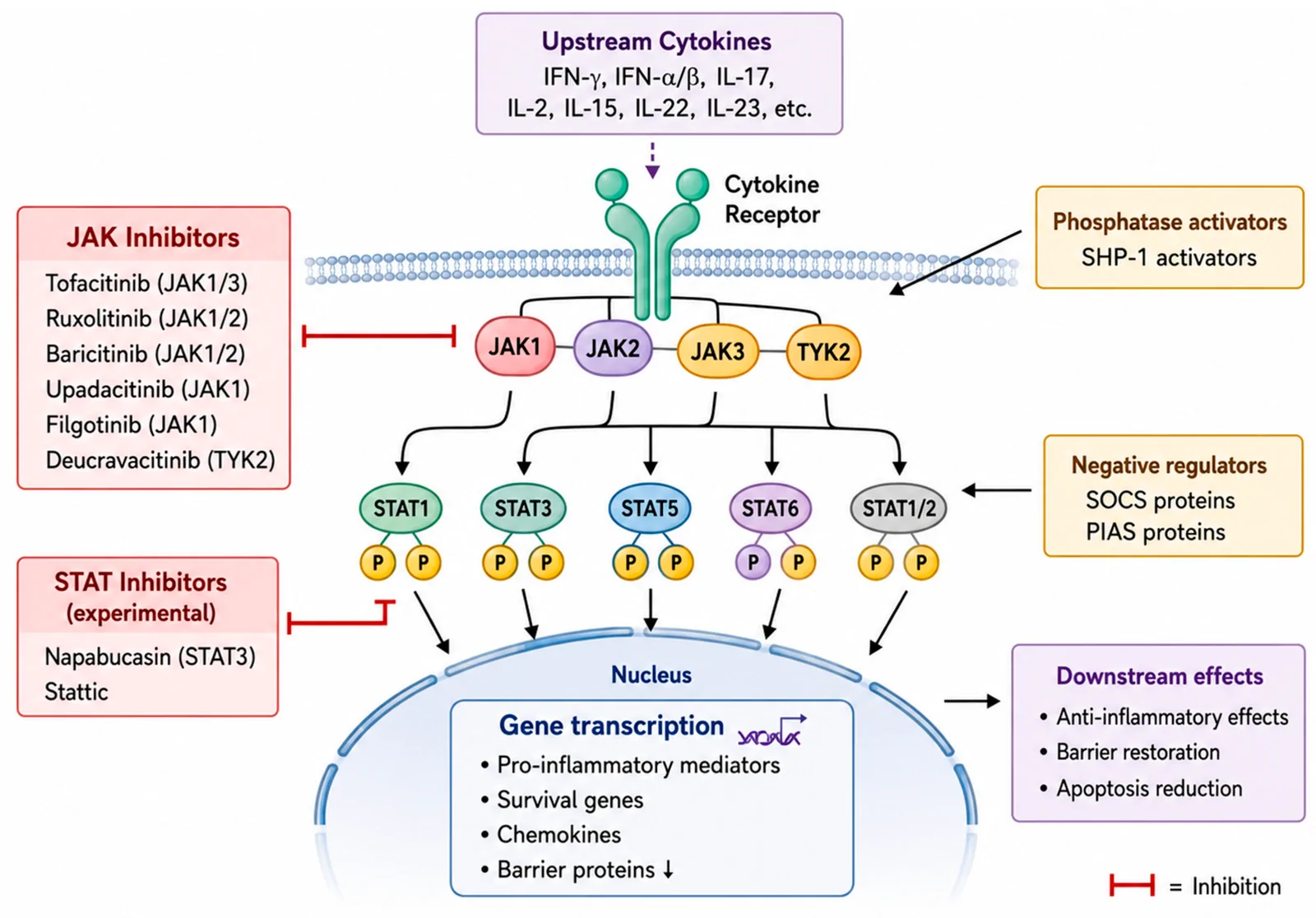
Targeting the JAK/STAT pathway in OLP: points of pharmacologic intervention. The figure maps clinically reported JAK/TYK2 inhibitors and experimental downstream strategies to different levels of the signaling network. Selectivity is relative and dose-dependent. IL-17 is included as part of the interacting inflammatory milieu rather than as a canonical JAK ligand; STAT inhibitors and phosphatase-directed approaches remain experimental in OLP.

Figure 3 places the reported and experimental inhibitors along the pathway. It should be interpreted as a pharmacologic concept map, not a treatment algorithm: direct OLP evidence exists mainly for JAK/TYK2 inhibitors, whereas STAT inhibitors and phosphatase-based strategies remain experimental and IL-17 is shown as part of the upstream inflammatory network rather than as a canonical JAK ligand [17,21]. Beyond kinase blockade, SHP-1 (PTPN6) represents a conceptually distinct negative-regulatory node within JAK/STAT signaling. As a protein tyrosine phosphatase, SHP-1 can promote signal termination by dephosphorylating activated signaling components; pharmacologic enhancement of SHP-1 activity could therefore, in principle, attenuate persistent cytokine-driven signaling [17,18]. OLP-specific transcriptomic evidence provides preliminary support for involvement of this regulatory node: an integrative mRNA/miRNA analysis identified PTPN6, the gene encoding SHP-1, among the genes significantly upregulated in OLP tissue compared with healthy oral mucosa [107]. This observation suggests that SHP-1-related phosphatase regulation may participate in the OLP inflammatory network, although its cellular source, functional activity and relationship with JAK/STAT signaling have not yet been directly established in OLP. Accordingly, pharmacologic enhancement of SHP-1 activity should currently be regarded as a hypothetical regulatory strategy rather than an established therapeutic approach.

## 9. A Precision-Pathway Agenda for OLP Research

### 9.1. Spatial Phospho-Endotyping and Causal Models

The next generation of OLP studies should move beyond bulk cytokine abundance and single-marker immunohistochemistry. Spatial phosphoproteomics, multiplex imaging and single-cell approaches could define which receptor–JAK–STAT combinations are active in basal keratinocytes, intraepithelial lymphocytes, subepithelial Trm cells and stromal populations within the same lesion [39,44,52]. This is particularly important at the edge of erosions, where histological architecture, cytokine exposure and barrier integrity may differ from the ulcer bed or clinically normal-appearing mucosa. Recent OLP-focused work further strengthens this spatial perspective: single-cell analysis identified SFRP2+Wnt5a+ fibroblasts interacting with CD8+ T cells and epithelial cells, while a 2026 review of T-cell immune synapses emphasized the organized cytotoxic interface between lymphocytes and basal keratinocytes [108,109]. A recent systematic review also found CCL5 upregulation across serum, T-lymphocyte and fibroblast compartments in OLP/OLL, but substantial methodological heterogeneity and risk of bias currently limit its value as a standalone biomarker [110].

Causality should then be tested in complementary systems. The IFN-γ-driven OLP-like mouse model closes a major preclinical gap, but organotypic human oral mucosal models and keratinocyte–T-cell co-cultures are needed to preserve species-specific epithelial biology and permit direct manipulation of E-cadherin/CD103, JAK/STAT modules and barrier proteins [43,44]. Patient-derived cells could also determine whether molecular endotypes observed in tissue are stable properties or transient states imposed by the local cytokine environment.

A key methodological priority is paired longitudinal sampling. The field currently has many cross-sectional observations but few datasets showing whether an IFN-high or STAT3/CCL5-rich state persists through flare, treatment response and relapse. Without that temporal dimension, an apparent endotype may simply capture disease activity at the moment of biopsy rather than a reproducible therapeutic phenotype.

### 9.2. Mechanism-Informed Trials and Locally Delivered Therapy

Future trials should compare mechanisms, not only drugs. One pragmatic design would stratify patients using an interferon/pSTAT1 signature, a STAT3–CCL5/barrier signature or a mixed phenotype, then test whether these states predict response to JAK1-predominant, JAK1/2 or TYK2-directed treatment [39,42,44,57,79]. Serial saliva and tissue biomarkers could distinguish pharmacodynamic target engagement from clinical improvement, while standardized REU or OLP-specific global assessments would anchor molecular changes to outcomes that matter to patients [46,86,87].

Local therapy deserves particular emphasis in this program. OLP is a surface-accessible disease, and many clinically significant lesions occupy a limited mucosal area. If adequate residence time and penetration can be achieved, local JAK inhibition could exploit the pathway’s tractability while minimizing systemic exposure and preserving the option of escalation for widespread or refractory disease [100,105]. Such trials should include pharmacokinetic sampling of saliva and mucosa, not merely clinical endpoints, because treatment failure may reflect insufficient tissue exposure rather than pathway irrelevance.

Ultimately, the most informative trial would connect four levels simultaneously: clinicopathologic phenotype, spatial pathway state, pharmacodynamic target engagement and clinical response. That design would test the central hypothesis of this review—that JAK/STAT signaling is useful not because it offers one universal target, but because it provides a structured way to decompose OLP into biologically testable inflammatory circuits.

## 10. Conclusions

Current evidence supports JAK/STAT signaling as a credible organizing framework for OLP immunobiology, but not as a single uniform pathogenic axis. The strongest causal evidence centers on IFN-γ–JAK1/JAK2–STAT1-mediated cytotoxic priming, while STAT3-centered circuits connect epithelial survival, metabolic stress, chemokine production, lymphocyte homing and barrier regulation. Common γ-chain and TYK2-dependent cytokines add additional layers that are biologically plausible but less completely mapped in oral lesions [42,43,44,59,76].

Conceptually, these data shift the relevant unit of disease from the isolated cytokine to the immune–epithelial interface. Spatially positioned Trm cells, keratinocytes, stromal cells and barrier structures form a reciprocal circuit, and JAK/STAT proteins route signals through that circuit in a cell- and context-dependent manner [44,52,55,65]. This framework helps explain why inhibitors with different kinase profiles can produce clinical responses without implying a single universal upstream driver.

Clinical translation is therefore promising but still immature. The repeated responses reported with JAK1-, JAK1/2-, JAK1/3-, JAK3/TEC- and TYK2-directed agents justify controlled OLP trials, yet the next step should be biomarker-guided rather than empiric [21,22,57,77,79,88,94,95,98,99]. Spatial phospho-endotyping, longitudinal biomarkers, organotypic human models and locally delivered therapy offer a realistic route toward that goal. If these elements are integrated prospectively, JAK/STAT biology may become more than a mechanistic explanation of chronic mucosal inflammation: it may provide a practical framework for precision treatment in OLP.

## Figures and Tables

**Table 1:** Cytokine–receptor–JAK–STAT modules relevant to oral lichen planus.

Cytokine/ Module	Principal Source/Context	Receptor/JAKs	Dominant STAT	Relevance in OLP	Evidence	Key References
IFN-γ	Th1/Tc1, CD8+ Trm, cytotoxic T cells	IFNGR1/2; JAK1/JAK2	STAT1	MHC-I induction, interferon genes, chemokines, cytotoxic priming	Strong functional LP; direct OLP human/model support	[42,43,51,52]
Type I IFN	Dendritic cells, keratinocytes	IFNAR1/2; JAK1/TYK2	STAT1/STAT2	Antigen presentation, interferon-stimulated genes	Direct innate-immune OLP evidence; limited causal testing	[15,40]
IL-6	Keratinocytes, T cells, myeloid/stromal cells	IL-6R/gp130; JAK1/JAK2/TYK2	STAT3	Epithelial survival/reprogramming; inflammatory amplification	Direct cytokine and STAT3 tissue evidence	[36,41,53]
IL-2/IL-15	Activated T cells; epithelial/myeloid sources	γc receptors; JAK1/JAK3	STAT5	T-cell expansion, survival, tissue persistence	Plausible; direct OLP kinase mapping limited	[34,54,55]
IL-21	Activated CD4+ and Tfh/Th17-like cells	IL-21R/γc; JAK1/JAK3	STAT3	T-cell differentiation and chronic inflammation	Strong LP rationale; OLP-specific direct data limited	[37,56]
IL-12	Dendritic cells, macrophages	IL-12Rβ1/β2; TYK2/JAK2	STAT4	Th1/Tc1 polarization and IFN-γ production	Mechanistically strong; supported by TYK2 therapeutic signal	[17,57]
IL-23	Dendritic cells, macrophages	IL-23R/IL-12Rβ1; JAK2/TYK2	STAT3/STAT4	Th17/Tc17 maintenance	Direct axis reported in OLP lesions	[49,50]
IL-22	Th17/Th22-related cells	IL-22R1/IL-10R2; JAK1/TYK2	STAT3	Epithelial stress, repair and proliferation	Preliminary OLP evidence	[58]
ROS-STAT3-CCL5	Metabolically stressed keratinocytes	Non-canonical redox-sensitive activation	STAT3	CCL5 production and T-cell recruitment	Direct OLP experimental evidence	[59]

**Table 2:** Published JAK/TYK2 inhibitor evidence most directly relevant to oral lichen planus.

Agent	Predominant Target	OLP Evidence	Representative Outcome	Main Limitation	Key References
Upadacitinib	JAK1-predominant	Multiple case reports and small case series, 2021–2026	Often rapid reduction in pain and erosive activity; several near-complete responses	Uncontrolled; heterogeneous diagnostic criteria and co-therapy	[88,89,90,91,92,93]
Abrocitinib	JAK1-predominant	Single direct OLP report	Marked improvement in refractory disease	Single-patient evidence	[94]
Baricitinib	JAK1/JAK2	Direct OLP reports; OLP-like model; mechanistic LP trial	Clinical improvement; suppression of IFN/cytotoxic program in cutaneous LP	No completed large OLP RCT	[43,73,95,96]
Ruxolitinib	JAK1/JAK2	OLP-like model; cutaneous LP prospective topical study	Preclinical OLP improvement; topical LP activity	Oral formulation/delivery not established	[43,100]
Tofacitinib	JAK1/JAK3, broader activity	Oral erosive LP case series; severe LP reports	Substantial symptom and lesion improvement in several patients	Broad activity; low-quality OLP evidence	[97,98,99]
Deucravacitinib	TYK2 allosteric	Case report and three-patient erosive OLP series	Re-epithelialization and reduction in lesion scores	Very small uncontrolled samples	[57,79]
Ritlecitinib	JAK3/TEC	Single erosive OLP case	Rapid symptomatic and clinical improvement	TEC-family activity; single case	[77]

## Data Availability

No new data were created or analyzed in this study. Data sharing is not applicable to this article.

## Abbreviations

The following abbreviations are used in this manuscript:

OLP	Oral lichen planus
JAK	Janus kinase
STAT	Signal transducer and activator of transcription
TYK2	Tyrosine kinase 2
IFN	Interferon
IL	Interleukin
Trm	Tissue-resident memory T cell
MHC-I	Major histocompatibility complex class I
SOCS	Suppressor of cytokine signaling
PIAS	Protein inhibitor of activated STAT
NF-κB	Nuclear factor kappa B
MAPK	Mitogen-activated protein kinase
PI3K	Phosphoinositide 3-kinase
AKT	Protein kinase B
mTOR	Mechanistic target of rapamycin
ROS	Reactive oxygen species
CCL5	C-C motif chemokine ligand 5
GLP-1	Glucagon-like peptide-1
REU	Reticulation/erythema/ulceration
TEC	Tyrosine kinase expressed in hepatocellular carcinoma
